# Aquaporin 4 inhibition alleviates myocardial ischemia-reperfusion injury by restraining cardiomyocyte pyroptosis

**DOI:** 10.1080/21655979.2021.1992332

**Published:** 2021-10-29

**Authors:** Qiong Jiang, Xianfeng Dong, Danqing Hu, Lejun Chen, Yukun Luo

**Affiliations:** a Department of Cardiology, Fujian Medical University Union Hospital, Fuzhou, Fujian, P.R. China; b Fujian Institute of Coronary Heart Disease, Fuzhou, Fujian, P.R. China; c Fujian Heart Medical Center, Fuzhou, Fujian, P.R. China

**Keywords:** Myocardial ischemia-reperfusion injury, pyroptosis, Aquaporin 4 (Aqp4)

## Abstract

Myocardial injury caused by ischemia-reperfusion is the main pathological manifestation of coronary artery disease (CAD), which is characterized by high mortality and morbidity. Thus, there’s an urgent need to develop efficacious strategies and elucidate the underlying mechanisms to prevent or alleviate myocardial ischemia-reperfusion injury to improve the clinical outcomes in patients. In this study, we took advantage of a typical myocardial cell line of mice (HL-1) and cultured with or without an aquaporin 4 inhibitor (TGN-20 denoted as AQP4i) under normal conditions (NC), ischemia (IS) and ischemia reperfusion (IR), respectively. The cytomorphology, ultrastructure, cell vitality and expression pattern of apoptotic proteins were verified with scanning electron microscope (SEM), immunofluorescence staining, flow cytometry, quantitative real-time PCR and western-blotting analysis, respectively. HL-1 under IS or IR condition revealed higher expression of Aquaporin 4 (Aqp4) compared to the NC group, whereas showed similarity in cytomorphology and ultrastructure. Aqp4 inhibition was sufficient to improve the apoptotic cells in HL-1 while showed minimal effects to the other cellular vitality. Furthermore, the expression pattern of apoptotic proteins and anti-apoptotic proteins together with proinflammatory factors in HL-1 was effectively rescued by Aqp4i treatment both at the mRNA level and protein level. Ischemia and ischemia reperfusion caused higher expression of Aqp4 and resultant increase of cardiomyocyte pyroptosis. Myocardial ischemia-reperfusion injury of HL-1 was effectively alleviated by Aqp4 and pyroptosis inhibition. Our findings provided new references for myocardial ischemia-reperfusion injury management via targeting Aqp4-mediated pyroptosis of cardiomyocyte.

## Introduction

Current studies have indicated myocardial injury as the leading cause of mortality in the month after surgery, which is one thousand times over anesthesia-associated intraoperative mortality [1,2]. Despite no safe and efficient prophylaxes for postoperative myocardial injury, yet there are strong correlations among ischemia-reperfusion (IR) and myocardial injury and thus reducing IR might therefore improve outcomes of the patients with myocardial injury [1–3].

Pyroptosis, a kind of programmed necrosis, has been recognized as a caspase-1-mediated monocyte death, which is a mechanistically and morphologically unique form of programmed cell death and involved in response to a certain number of bacterial insults [4,5]. Mechanistically, pyroptosis is originally driven by two major signaling pathways including the one mediated by caspase-4/5/11 and the other by caspase-1 [6–8]. Recent studies have shown that pyroptosis is implicated in several cardiovascular diseases such as myocardial infarction, atherosclerosis, reperfusion injury, diabetic cardiomyopathy and myocarditis [6,9]. However, the detailed information of the function and mechanism of pyroptosis as well as the association with cardiovascular diseases including myocardial ischemia-reperfusion injury still requires thorough disclosure [10].

Aquaporin 4 (AQP4), a water transporting protein, is involved in a series of physiological processes, including glymphatic solute transport, synaptic plasticity, learning, memory [11,12]. It’s noteworthy that Zeng and the colleagues verified that AQP4 knockout could aggravate ischemia-reperfusion (IR) injury in mice, which provided direct evidence for further dissecting the crucial role of AQP4 in the pathogenesis of IR injury and a novel option for myocardial injury administration [13]. However, the detailed function together with the underlying mechanism of AQP4 during IR injury are still far from satisfaction.

Therefore, on the basis of the aforementioned studies, we hypothesized that AQP4 might function a critical role in ischemia-reperfusion (IR) injury *in vitro* and might be a potential candidate for IR management. In this study, we took advantage of the mouse cardiomyocyte cell line and the classical myocardial IR injury model to investigate the function and mechanism of AQP4-mediated pyroptosis. Our data revealed the pivotal role of AQP4 in alleviating the impairment of IR-associated cellular viability by inhibiting pyroptosis rather than apoptosis. Additionally, we also noticed that AQP4 inhibition revealed minimal effect to the cytomorphology of mouse cardiomyocytes.

## Materials and methods

### Cell culture

The HL-1 mouse cardiomyocyte cell line was purchased from American Type Culture Collection (ATCC) and cultured according to the manufacture’s introductions. The cells were cultured in DMEM basal medium (Hyclone, SH30022.01) supplemented with 10% FBS (Gibco, 10099141), 1% penicillin and streptomycin (P/S) under normoxia (20% v/v) or hypoxia (1% v/v) conditions in 37°C, 5% CO_2_. For mimicking the ischemia reperfusion injury (IRI), the HL-1 cells were cultured in glucose-free DMEM basal medium (Gibco) without FBS addition for 1 hr, then the medium was changed with high glucose DMEM basal medium (Gibco) and 10% FBS addition for 2 hrs.

### Flow cytometry (FCM) assay

The HL-1 cells in the indicated groups were dissociated into single cells and incubated with the indicated antibodies against Annexin-V (Annexin V-FITC), 7AAD and PI in 0.2% BSA for 30 min in dark. Then, the cells were washed with 1× PBS for twice and turned to FCM assay by using the FACS Canto II (BD) as previously described [14,15].

### Western-blotting assay

The expression levels of the proteins in HL-1 cells under the indicated conductions were quantified by utilizing the western-blotting assay and the gray scanning method as previously reported [16]. In details, the HL-1 cells were washed with 1× PBS for twice and lysed by RIPA buffer (meilunbio, MA0151) with protease inhibitor Cocktail (EDTA-Free, 100×, in DMSO, MedChemExpress) and PMSF (Kaiji, KGP610) addition. The target protein bands were separated by SDS-PAGE electrophoresis (Beyotime Biotech, P0015L) and then transferred into NC membrane (Merck, HATF0010). After that, the protein bands in the membrane were labeled with the primary antibodies such as AQP4 (proteintech, 16473-1-AP) and GAPDH (Abcam, ab8245) and the HRP-conjugated secondary antibodies (proteintech, SA00006-4). Finally, the bands were developed by using the ECL kit (ThermoFisher) and gray scanning (image lab).

### Enzyme activity detection

The activity of Caspase-1 was quantified with the commercial kit (Dalian Meilun Biotech. Co., Ltd, MA0327-L) and the BCA protein assay kit (Dalian Meilun Biotech. Co., Ltd, MA0079). Briefly, the HL-1 cells in the control groups and the experimental groups were harvested by centrifugation and lysed by lysate for 15 min under ice bath. The supernatant was collected for the detection of Caspase-1 activity according to the manufacture’s introductions.

### Enzyme-linked immunosorbent assay (ELISA)

The concentrations of the secreted interleukins (ILs) by HL-1 cell lines such as IL-1β (JL18442, Jianglai Biotech.) and IL-8 (JL20271, Jianglai Biotech.) were quantified with the indicated kits according to the manufacture’s introductions. In brief, the kit was placed at room temperature (RT) for 20 min for preparation. 100 μl HRP-conjugated secondary antibodies were added to 50 μl the standard substance or protein samples in the wells of the plates, and then incubated at 37°C for 1 hr. After that, the wells were washed with 350 μl washing buffer for five times and incubated with the 50 μl A/B substrates in dark for 15 min. Finally, the concentrations of the secreted ILs were quantified under 450 nm on the basis of OD value.

### Quantitative real-time PCR (qRT-PCR) assay

The expression levels of the indicated mRNAs were quantified by the qRT-PCR assay as recently described [14,17]. Briefly, the HL-1 cells under the indicated treatments were lysed with the TRIZol reagent (ThermoFisher) and the total mRNAs were synthesized into cDNA by using the reverse transcription kit (ThermoFisher). The cDNAs were used for the detection of the expression levels of the indicated genes (e.g., GSDMD, Beclin-1, LC3-II, Bcl-2, Bax, caspase-1, caspase-3, caspase-4, caspase-5, caspase-11) by using the ABI-7500 qRT-PCR instrument and the standard 2^−ΔΔct^ method.

### Immunofluorescent staining

The immunofluorescent staining of the indicated proteins in HL-1 cells in the aforementioned groups were conducted as previously reported with several modifications [18–20]. In details, the HL-1 cells were washed with 1× PBS for twice and fixed with 4% PFA for 15 min. Then, the cells were permeabilizated with 0.2% Triton X-100 for 45 min and stained with rabbit-anti-mouse GSDMD for 24 hr and stained with donkey-anti-rabbit Alexa Fluor 594-conjugated secondary antibody for 45 min. DAPI was used for nucleus staining as previously reported [21].

### JC-1 staining

The JC-1 staining was conducted with the mitochondrial membrane potential detection kit (MA0338, Meilun, China) according to the manufactures’ instructions. In details, the dyeing working solution was prepared by diluting the JC-1 (200×) buffer with H_2_O (Thermo Fisher, USA). The HL-1 cells in the indicated groups were washed with 1× PBS and treated with 10 μM CCCP for 20 min. The cells were observed and recorded under the fluorescence microscope (VMF20A, MicroDemo, USA).

### Statistical analysis

All the statistical analyses were performed by using the GraphPad Prism 6.0 Software (GraphPad, USA) as reported before [18,21–23]. In brief, the unpaired t test and one-way ANOVA test were used for analyzing the two different unpaired groups and multiple unpaired groups, respectively. All the data were shown as Mean±SEM (N = 3 independent experiments). *, P < 0.05; **, P < 0.01; ***, P < 0.001; NS, not significant.

## Results

### AQP4 inhibition showed minimal effect to the cytomorphology of mouse cardiomyocytes

As mentioned above, despite the in vivo study by Zeng et al has indicated the critical role of AQP4 in the pathogenesis of IR injury, yet the direct evidence of AQP4 inhibition in vitro as well as the detailed information of the underlying molecular mechanism are far from satisfaction [13]. Therewith, we raised the presumption that AQP4 could play a critical role in IR injury *in vitro* as well and held the potential to act as an important candidate for IR management.

**Figure 1.**
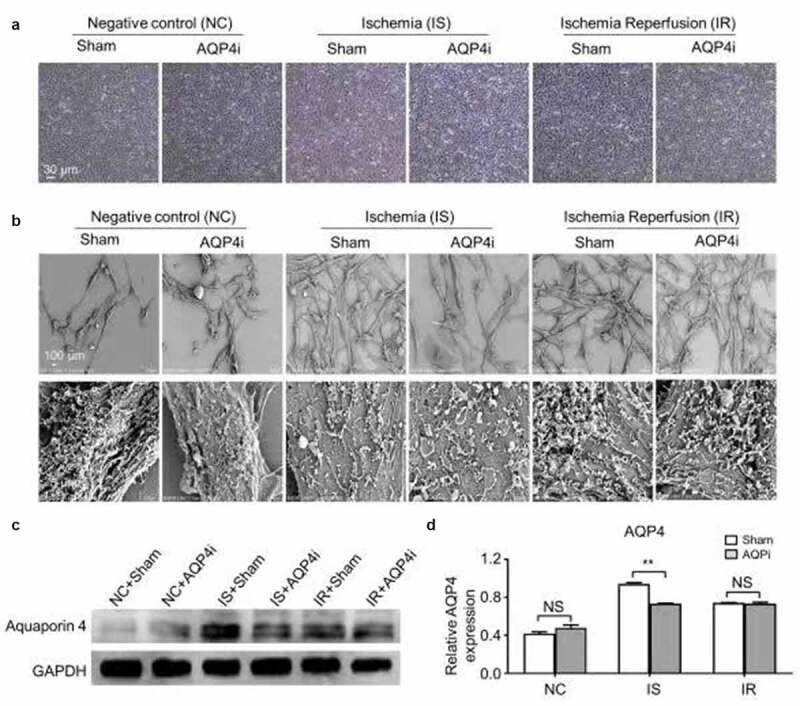
The cytomorphology of HL-1 cells and AQP4 expression with AQP4 inhibition

Meanwhile, current advances have indicated the involvement of ischemia-reperfusion in the occurrence of myocardial injury, yet the underlying mechanisms as well as the potential interventions are still obscure. For the purpose, we took advantage of the HL-1 mouse cardiomyocyte cell line to explore the function of Aquaporin 4 (AQP4) in the ischemia-reperfusion model in vitro. HL-1 cells with AQP4 inhibitor (denoted as AQP4i) treatment or not (denoted as Sham) under normoxia (20% v/v, negative control, denoted as NC), hypoxia (1% v/v, ischemia, denoted as IS) and ischemia-reperfusion (1% v/v for 2 hrs and 20% v/v for 1 hr, denoted as IR) conditions showed similarities in cytomorphology (Figure 1(a)), which was confirmed by the microstructural and ultrastructural analyses on the basis of scanning electron microscope (SEM) (Figure 1(b)). Furthermore, as shown by the western-blotting analysis, HL-1 cells with minimal expression of AQP4 in HL-1 cells in the NC groups (NC+Sham, NC+AQP4i), whereas with higher level of AQP4 expression under IS (IS+Sham, IS+AQP4i) and IR (IR+Sham, IR+AQP4i) conditions, and in particular, the IS+Sham group with the highest level of AQP4 expression (Figure 1(c)). Simultaneously, we found the addition of AQP4i was sufficient to inhibit AQP4 expression in HL-1 cells under IS conditions (Figure 1(c–d)).

(a). Phase contrast images of HL-1 cells without (Sham) and with AQP4i (AQP4i) treatment in the indicated groups. Scale bar = 30 μm. (b). Ultrastructure images of HL-1 cells by utilizing electron microscopy without (Sham) and with AQP4i (AQP4i) treatment in the indicated groups. Scale bar = 100 μm. (c). Western-blotting assay of Aquaporin 4 (AQP4) and GAPDG in HL-1 cells in the aforementioned groups. (d). Quantitative analysis of Aquaporin 4 (AQP4) protein expression in the aforementioned groups. **, P < 0.01; NS, not significant.

### AQP4 inhibition effectively alleviated the impairment of ischemia-reperfusion associated cellular viability

**Figure 2.**
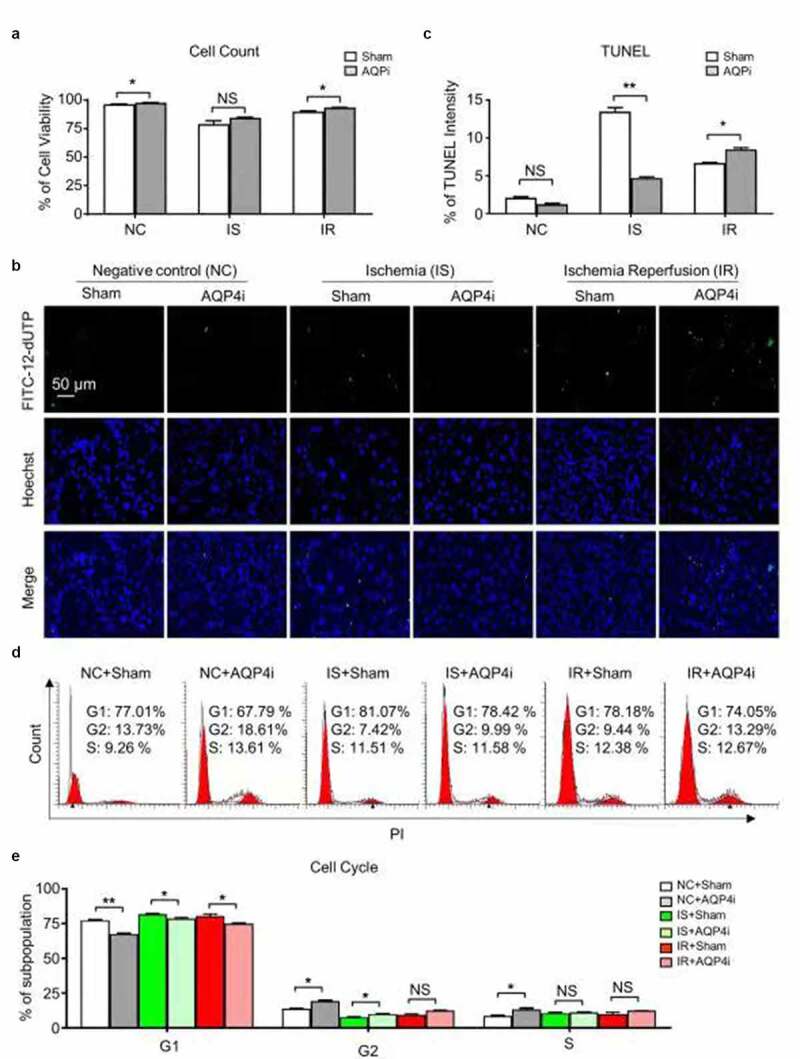
The influence of AQP4 inhibition upon cellular viability of HL-1 cells

To further explore the potential influence of AQP4i to the cellular vitality of HL-1 in the ischemia-reperfusion model, we initially compared the NC, IS and IR groups and found that AQP4i showed minimal effect to the total cell number (Figure 2(a)). Interestingly, the immunofluorescence-based TUNEL intensity assay visually reflected the inhibitory effect of AQP4i to the apoptosis of HL-1 mouse cardiomyocyte cell line under IS and IR conditions (Figure 2(b,c)). However, according to the subpopulation analysis of cell cycle, we didn’t observe the obvious effect of AQP4i upon HL-1 cell division (Figure 2(d,e)). Taken together, AQP4 inhibition effectively alleviated the apoptosis rather than cell cycle and the resultant impairment of ischemia-reperfusion in HL-1 cells.

(a). The cell viability of HL-1 cells without (Sham) and with AQP4i (AQP4i) treatment in the indicated groups. *, P < 0.05; NS, not significant. (b,c). Evaluation of the inhibitory effect of AQP4i to the apoptosis of HL-1 cells by immunofluorescence-based TUNEL intensity assay. Scale bar = 50 μm. D-E. FCM diagram (d) and statistical analysis (e) of the subpopulations of cell cycle in the aforementioned groups. *, P < 0.05; **, P < 0.01; NS, not significant.

### The alleviative effect of AQP4i upon ischemia-reperfusion was apoptosis-independent

**Figure 3.**
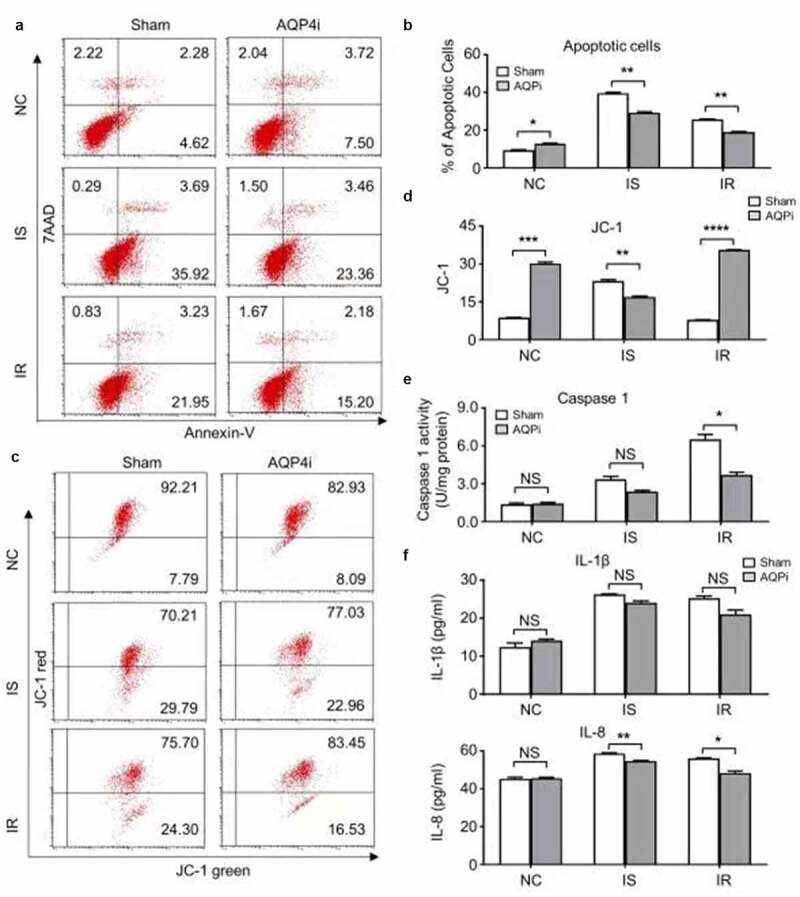
The alleviative effect of AQP4i upon ischemia-reperfusion was apoptosis-independent

Having indicated the inhibitory effect of AQP4i upon TUNEL intensity, we are further curious about the underlying mechanism of AQP4 in the HL-1 cell-based ischemia-reperfusion model. Therefore, we initially detected the apoptotic population of HL-1 cells in the indicated groups with or without AQP4i treatment. Despite the moderate increase of apoptotic cells in the IS and IR groups compared with that in the NC groups, yet we didn’t notice the remarkable influence of AQP4i treatment among the indicated groups (Figure 3(a,b)). Simultaneously, even though the percentage of HL-1 cells with apoptosis-associated JC-1 expression in the NC groups were higher than those in the IS and IR groups, yet there were no significant differences in the corresponding groups with or without AQP4i treatment as well (Figure 3(c,d)). Distinguish from the apoptosis-associated indicators including Annexin Ⅴ7, AAD and JC-1, the pyroptosis-associated Caspase 1 activity in the IS and IR groups with AQP4i treatment was decreased when compared with the corresponding Sham ones (IS+Sham, IR+Sham), respectively (Figure 3(e)). Similarly, the inhibitory effect of AQP4i treatment upon the expression of IL-8 rather thanIL-1β was also confirmed in the HL-1 mouse cardiomyocyte cell line (Figure 3(f)). Collectively, our data suggested the alleviative effects of AQP4i upon the impairment of ischemia-reperfusion via inhibiting pyroptosis rather than apoptosis.

(a,b). FCM diagram (A) and statistical analysis (B) of the apoptotic HL-1 cells in the aforementioned groups without (Sham) and with AQP4i (AQP4i) treatment. *, P < 0.05; **, P < 0.01. C-D. FCM diagram (c) and statistical analysis (d) of the JC-1 positive cells in the aforementioned groups. **, P < 0.01; ***, P < 0.001; ****, P < 0.0001. E-F. The pyroptosis-associated Caspase 1 activity (e) and the concentrations of cytokines including IL-1β and IL-8 (f) in the supernatant of HL-1 cells in the aforementioned groups. *, P < 0.05; **, P < 0.01; NS, not significant.

### AQP4i relived ischemia-reperfusion principally by inhibiting pyroptosis

**Figure 4.**
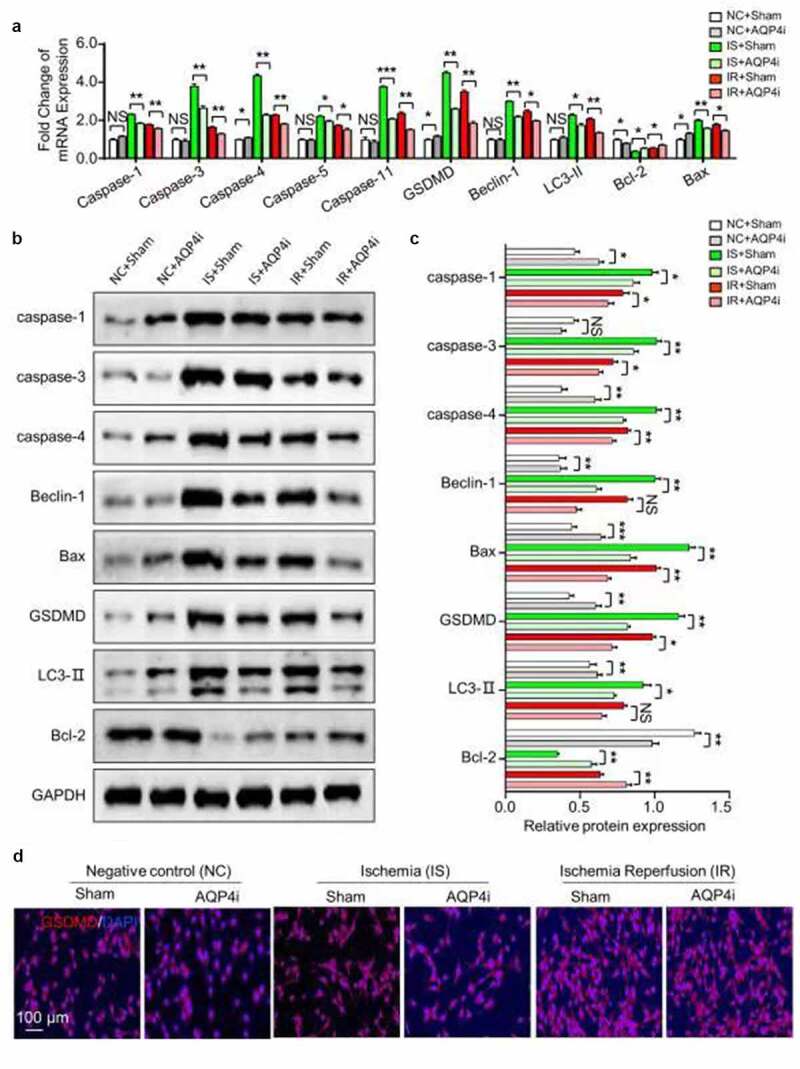
The inhibitory effect of AQP4i upon pyroptosis in HL-1 cells

Aiming to further clarify the alleviative effect and underlying mechanism of AQP4i treatment upon ischemia-reperfusion, we systematically examined the expression pattern of pyroptosis-associated genes both at the mRNA and protein level. On the one hand, most of the pyroptosis-associated genes including Caspase family members (Caspase 1, Caspase 3, Caspase 4, Caspase 5, Caspase 11), GSDMD, Beclin-1 and LC3-II showed consistent trends of mRNA expression, which were collectively down-regulated in the IS+AQP4i and IR+AQP4i groups compared with those in the IS+Sham and IR+Sham groups, respectively (Figure 4(a)). On the other hand, by utilizing the western-blotting assay, we found that the expression levels of the aforementioned genes showed similarities in corresponding protein expression (Figure 4(b,c)). For instance, with the aid of immunofluorescent staining, we further confirmed the inhibitory effect of AQP4i treatment upon GSDGM expression in HL-1 cells in the IS and IR groups (Figure 4(d)). Taken together, we identified the alleviative effect of AQP4i upon ischemia-reperfusion via a pyroptosis-associated approach.

(a). Quantitative analysis of the pyroptosis-associated biomarker expression in HL-1 cells without (Sham) and with AQP4i (AQP4i) treatment by utilizing qRT-PCR. *, P < 0.05; **, P < 0.01; NS, not significant. (b). Western-blotting assay of the pyroptosis-associated biomarker and GAPDG expression in HL-1 cells in the aforementioned groups. GAPDH was used as a loading control. (c). Quantitative analysis of the pyroptosis-associated biomarker expression in the aforementioned groups. *, P < 0.05; **, P < 0.01; NS, not significant. (d). Immunofluorescent staining of the pyroptosis-associated biomarker GSDMD expression in HL-1 cells in the aforementioned groups. DAPI was used for nuclear staining. Scale bar = 100 μm.

## Discussion

Generally, myocardial ischemia-reperfusion injury represents a mode of action that might has serious impact upon the outcomes and care of inpatients with myocardial ischemia [2]. Even though a mass of preclinical studies has demonstrated the effective protection from IR injury in animal disease models, yet translation into clinical practice is largely less successful [2,3]. In this study, we verified the detailed function and mechanism of AQP4-mediated pyroptosis in the in vitro mouse myocardial IR model administration. Interestingly, we found that AQP4 inhibition exhibited minimal effect to the cytomorphology of the HL-1 mouse cardiomyocytes but effectively alleviated the impairment of ischemia-reperfusion associated cellular viability instead, which mainly functions via a gasdermin (GSDMD)-dependent pyroptosis approach. Our findings add new references for Aqp4-mediated myocardial ischemia-reperfusion injury management in future.

Generally, pyroptosis features the gasdermin family-mediated cell lysis caused by membrane pore formation, together with the release of proinflammatory intracellular contents such as IL-1β, IL-18 and HMGB1 [6,8]. Distinguish from apoptosis, pyroptosis allows the release of a series of immunogenic cellular content such as inflammatory cytokines (e.g., interleukin-1β) and damage-associated molecular patterns (DAMPs) [24]. In the past decade, many studies have investigated the characteristics of pyroptosis in cardiovascular disease (CVDs), which further resulted in the development of therapeutic strategies based on the regulation of pyroptosis [6,24].

Besides the aforementioned function in ion and water homeostasis, AQP4 has also been demonstrated to be involved in a number of acute and chronic cerebral pathologies such as the development of neuromyelitis optica, amyotrophic lateral sclerosis (ALS), Alzheimer’s disease, Parkinson’s disease and autoimmune neurodegenerative diseases [25–28]. For instance, Zou et al found that the upregulation of AQP4 was noted in correlation with vessels surrounded by swollen astrocytic processes, whereas AQP4 suppression is adequate for the improvement of motor function in ALS [28]. Herein, we further verified the detailed function of AQP4 in the mouse model of myocardial ischemia-reperfusion injury, which mainly via a mechanism of GSDMD-dependent pyroptosis approach. Additionally, we observed that AQPi treatment didn’t influence the secretion of Caspase 1 activity in the supernatant of the NC group but did influence the expression in HL-1 cells in the NC groups instead.

It’s noteworthy that recent updates have also suggested the pivotal role of other genes including Connexin43 (CX43) in the heart development, myocardial function and coordination of electrically coupled cardiomyocytes activities, and in particular, the connection between myocardial ischemia-reperfusion and Cx43 has become the focus of the field [29]. Simultaneously, Raza and the colleagues identified the Sphingosine 1-phosphate, a pivotal metabolite of sphingolipids, in myocardial ischemia and reperfusion injury by upregulating RISK-SAFE pro-survival cascades [30]. However, many of the mechanisms are still obscure and require in-depth research. Overall, our study lighted the possibility and new option of myocardial ischemia-reperfusion injury management on the basis of Aqp4-mediated pyroptosis in future.

## Conclusion

Overall, we verified the critical role of Aquaporin 4 inhibition in alleviating the myocardial ischemia-reperfusion injury, which mainly functioned via restraining cardiomyocyte pyroptosis rather than influencing apoptosis. Collectively, our studies provided overwhelming new references for myocardial IR injury management by targeting the Aquaporin 4-mediated pyroptosis of cardiomyocyte.
